# Altered enzyme expression in "differentiated" murine neuroblastoma cells.

**DOI:** 10.1038/bjc.1976.159

**Published:** 1976-09

**Authors:** N. Prasad, R. Prasad

## Abstract

**Images:**


*Br. J. Cancer* (1976) **34**, 249

### ALTERED ENZYME EXPRESSION IN " DIFFERENTIATED " MURINE NEUROBLASTOMA CELLS

N. PRASAD AND R. PRASAD

*From the Departments of Radiology and Obstetrics and Gynecology, Baylor College of Medicine, Houston, Texas* 77030, *and the Radiobiology Research Laboratory, V. A. Hospital, Houston, Texas* 77211

Received 11 March 1976  Accepted 20 May 1976

**Summary.—Out of 17 enzymes studied, only 9 were detectable by starch gel electrophoresis in mouse neuroblastoma cells in culture. Prostaglandin $E_1$ ($PGE_1$) and 4(-3-butoxy-4-methoxybenzyl)-2-imidazolidinone (R020-1724), a specific inhibitor of cAMP phosphodiesterase, were used to induce " differentiation ". Lactate and 6-phosphogluconate dehydrogenases and adenylate kinase were expressed as single bands in untreated neuroblastoma and induced " differentiated " cells, but the electrophoretic mobility of these enzymes in $PGE_1$-treated cells was slower than that in malignant and R020-1724-treated cells. Three bands of glucose 6-phosphate dehydrogenase were detectable in $PGE_1$-treated cells, whereas the R020-1724-treated cells had two bands and the untreated neuroblastoma cells had only one band. Aldolase was also expressed as a single band; however, the activity of this enzyme was much higher in $PGE_1$-treated cells, whereas the activity was barely detectable for R020-1724-treated and untreated neuroblastoma cells. Some of the enzymes which are present *in vivo* are absent *in vitro*. Alkaline phosphatase is present in brain but is absent in neuroblastoma cells *in vivo* and *in vitro*. Two bands each of triose phosphate isomerase, fumarase and aldolase are present in brain, but only one band of these enzymes is present in neuroblastoma cells. Although $PGE_1$ and R020-1724 induce many differentiated functions in neuroblastoma cells in a similar manner, $PGE_1$ appears to change characteristically the expression of several enzymes.**

AN ELEVATION of the intracellular level of cAMP in neuroblastoma cells in culture induces many differentiated functions which are characteristic of mature neurons. These include formation of long neurites, (Prasad and Hsie, 1971) increase in size of soma and nucleus associated with an increase in total RNA (Augusti-Tocco, *et al.*, 1973; Prasad *et al.*, 1973) blockade of cells in $G_1$-phase of cell cycle, (Prasad *et al.*, 1973), increase in activities of tyrosine hydroxylase, (Richelson, 1973; Waymire, Weiner and Prasad, 1972) choline acetyltransferase (Prasad and Mandal, 1973) and acetylcholinesterase, (Furmanski, Silverman and Lubin, 1971; Blume *et al.*, 1970), loss of tumourigenicity (Prasad, 1972) and increase in sensitivity of adenylate cyclase to catecholamines (Prasad and Kumar, 1974). We have shown that the muscle-type lactate dehydrogenase (LDH-5) which prevails in embryonic tissue (Wolf and Engel, 1972) is present in neuroblastoma cells, but absent from mouse brain tissue (Prasad, Prasad and Prasad, 1973), indicating the re-expression of an embryonic feature during malignant transformation. In addition, it has been reported (Bondy, Prasad and Purdy, 1974) that the amount of poly-adenylic-(A)-containing cytoplasmic RNA is greater in cAMP-induced " differentiated " neuroblastoma cells than that in malignant cells. These data indicate that there may be an alteration in the expression of genetic information during the time of " differentiation " of neuroblastoma cells in culture. Therefore, we have investigated the activities of 17 enzymes in malignant and " differentiated " cells. We now report the following: (a) several of the enzymes which are present in brain are absent in neuroblastoma cells *in vivo* and *in vitro*; (b) prostaglandin($PGE_1$)-induced changes in the expression of enzyme pattern are different from those induced by 4-(-3-butoxy-4-methoxybenzyl)-2-imidazolidinone (R020-1724), an inhibitor of cAMP phosphodiesterase.

### MATERIALS AND METHODS

*Cell culture.*—The procedure for culturing mouse neuroblastoma cells has been previously described by Prasad and Hsie (1971). Previously defined neuroblastoma clone $NBA_2$ (Bondy *et al.*, 1974) was used in this study. Neuroblastoma cells contain tyrosine hydroxylase and acetylcholinesterase activities, but have no choline acetyltransferase activity. The average doubling time is about 18 h. The procedures for making solutions were previously described (Prasad and Hsie, 1971). Prostaglandin $E_1$, a stimulator of adenylate cyclase, and 4-(-3-butoxy-4-methoxybenzyl)-2-imidazolidinone (R020-1724), a specific inhibitor of cAMP phosphodiesterase (Sheppard, Wiggan and Tsien, 1972) induce many differentiated functions in these cells (Prasad and Kumar, 1974). Therefore, these agents were used to induce differentiation in neuroblastoma cells in culture. $PGE_1$ (10 $\mu$g/ml) and R020-1724 (200 $\mu$g/ml) were added separately 24 h after plating the cells ($5 \times 10^5$) in large Falcon plastic flasks. The drug and medium were changed every day and cells were removed from the flask surface, using Viokase solution, 3 days after treatment. The cells were washed twice with phosphate buffer solution.

To prepare the cell homogenate (*in vitro* study) the cells were harvested by centrifugation and washed twice with 0·9% saline. The cells were then resuspended in deionized distilled water to make a concentration of $5 \times 10^6$ cells/ml. The cell membranes were then disrupted by alternate freezing and thawing. The supernatant obtained after the centrifugation at 10,000 *g* was used for the electrophoresis.

Tumours were produced in male A/J mice by injecting cells of clones $NBA_2$ (Bondy *et al.*, 1974). The tumour was allowed to grow for 15 days. Crude tissue extracts (*in vivo* study) of tumour, brain and kidney were prepared as described by Prasad, Prasad and Tevethia (1972). All the samples were subjected to vertical starch gel electrophoresis. The protein concentration of each of these samples ranged from 1·8 to 2·0 mg/ml. The buffer systems used for different enzymes were the same as previously described (Prasad *et al.*, 1974). Gel slices were stained for the following enzymes by the routine methods (Shaw and Prasad, 1970): dehydrogenases of lactate (LDH), malate (MDH), glutamate (GDH), glucose-6 phosphate (G6PD), 6-phosphogluconate (6PGD), hexose-6 phosphate (H6PD), and isocitrate (IDH), α-esterase, fumarase, phosphoglucomutase (PGM), aldolase, adenylate kinase (AK), acid and alkaline phosphatases, hexokinase (HK) and triose-phosphate isomerase (TPI).

### RESULTS AND DISCUSSION

### In vitro *system*

The Table summarizes the results of our study. Out of 17 enzymes studied, only 9 were detectable by starch gel electrophoresis in neuroblastoma cells in culture. Five of these 9 enzymes showed electrophoretic variation. LDH and 6PGD and AK were expressed as a single band in malignant cAMP-induced and " differentiated " cells, but the electrophoretic mobility of these enzymes in $PGE_1$-treated cells was slower than in control (untreated neuroblastoma) cells, R020-1724-treated cells and tumour tissue extract. Fig. 1 shows the electrophoretic pattern of 6PGD. The pattern of G6PD was interesting. Three bands of this enzyme were detectable in $PGE_1$-treated cells whereas the R020-1724-treated cells had two bands. The control cells as well as tumour tissue extract had only one band (Fig. 2). Aldolase was also expressed as a single band; the activity was much higher in $PGE_1$-treated cells, whereas the activity was barely detectable for R020-1724-treated and control cells.

TABLE.—*Expression of Enzymes of $PGE_1$- and R020-1724-treated Murine Neuroblastoma Cells in Culture and in Brain and Tumour Tissues*

| | | *In vitro* system | | | | *In vivo* system | | | |
|---|---|---|---|---|---|---|---|---|---|
| No. | Enzyme | R020-1724-treated cells | Control cells | $PGE_1$-treated cells | Presence of electrophoretic variants | Tumour | Brain | Kidney | Presence of electrophoretic variants |
| 1 | IDH | — | — | — | — | — | — | + | — |
| 2 | Alkaline phosphatase | — | — | — | — | — | + | + | + |
| 3 | Fumerase | — | — | — | — | + | + | + | + |
| 4 | αGPD | — | — | — | — | + | + | N.D. | — |
| 5 | GDH | — | — | — | — | + | + | + | — |
| 6 | α-esterase | — | — | — | — | + | + | + | + |
| 7 | HK | — | — | — | — | — | — | — | — |
| 8 | H6PD | — | — | — | — | — | — | — | — |
| 9 | Acid phosphatase | + | + | + | — | + | + | + | + |
| 10 | PGM | + | + | + | — | + | + | + | + |
| 11 | Aldolase | + | + | + | + | + | + | N.D. | + |
| 12 | AK | + | + | + | + | + | + | N.D. | — |
| 13 | MDH | + | + | + | — | + | + | + | — |
| 14 | LDH | + | + | + | + | + | + | + | + |
| 15 | G6PD | + | + | + | + | + | + | + | — |
| 16 | 6PGD | + | + | + | + | + | + | + | — |
| 17 | TPI | + | + | + | — | + | + | N.D. | + |

IDH Isocitrate dehydrogenase

αGPD α-glycerophosphate dehydrogenase

GDH Glutamate dehydrogenase

HK Hexokinase

H6PD Hexose-6-phosphate dehydrogenase

PGM Phosphoglucomutase

AK Adenylate kinase

MDH Malate dehydrogenase

LDH Lactate dehydrogenase

G6PD Glucose-6-phosphate dehydrogenase

6PGD 6-phosphogluconate dehydrogenase

TPI Triose phosphate isomerase

\+ Present

— Absent

N.D. Not done

FIG. 1.—Starch gel electrophoretic patterns of 6PGD from differentiated neuroblastoma cells *in vitro* (o, origin). Samples are: (a) tumour tissue extract, (b) control cells, (c) R020-1724-treated cells, (d) $PGE_1$-treated cells.

FIG. 2.—Starch gel electrophoretic patterns of G6PD from differentiated neuroblastoma cells *in vitro* (o, origin). Samples are: (a) $PGE_1$-treated cells, (b) R020-1724-treated cells, (c) control cells, (d) tumour tissue extract.

In vivo *system*

HK and H6PD were the only 2 enzymes which were not detectable in any tissue (Table). IDH was present in kidney but absent in brain and tumour tissue. Eight of the enzymes showed electrophoretic variation. A single band of AK, TPI, G6PD, fumarase and 6PGD hydrogenase was present in tumour tissue. In the samples of brain tissue, however, 2 bands of aldolase, fumarase and TPI were present. Two bands of fumarase and TPI were also present in kidney tissue.

The result shows that some of the enzymes which are present *in vivo* are absent *in vitro* (Nos. 1–6 of Table). Alkaline phosphate is present in brain and kidney tissue but absent in neuroblastoma cells *in vivo* and *in vitro*. We do not know if the absence of enzymes from the tumour tissue reflects an embryonic or a malignant characteristic or the lack of glial components of the nervous tissue. The presence of 2 bands of TPI, fumarase and aldolase in brain, and the presence of single bands of these 3 enzymes in tumours, indicate either the loss of tissue-specific isozyme during malignant transformation, or that the 2nd bands of these enzymes are of glial origin and therefore may be demonstrated in brain but not in pure neuronal culture. Therefore, any change or lack of it in the expression of the enzyme pattern in " differentiated " cells when compared to brain tissue may or may not be the differentiated functions of mature neurons.

Although $PGE_1$ and R020-1724 induce many differentiated functions in neuroblastoma cells in a similar manner, (Furmanski, Silverman and Lubin, 1971, Prasad and Kumar, 1974) $PGE_1$ appears to change characteristically the expression of AK and G6PD, 6PGD and LDH. From these data it is clear that $PGE_1$-treated neuroblastoma cells in culture express different enzyme patterns from those cells which were treated with R020-1724. Previous studies have shown that many differentiated functions in neuroblastoma cells are induced both by $PGE_1$ and R020-1724 (Furmanski *et al.*, 1971; Prasad and Kumar, 1974). However, these agents produce different types of membrane changes (Prasad and Sheppard, 1972). For example, R020-1724-induced " differentiated " neuroblastoma cells do not agglutinate, whereas $PGE_1$-induced " differentiated " cells agglutinate similarly to malignant cells. Our present study shows that certain enzymatic expressions of $PGE_1$-treated cells are also different from those of R020-1724-treated cells. These data suggest that the expression of enzyme patterns in $PGE_1$-treated cells is different from that of R020-1724-treated cells, although both agents increase intracellular levels of cAMP and induce many differentiated functions in a similar manner (Furmanski *et al.*, 1971; Gilman and Nirenberg, 1971; Richelson, 1973).

This work was supported in part by the United States Public Health Service Grant CA-10893.

REFERENCES

AUGUSTI-TOCCO, G., PARISI, E., ZUCCO, F., CASOLA, L. & ROMANO, M. (1973) Biochemical Characterization of a Clonal Line of Neuroblastoma. *Tissue Culture of the Nervous System*. Ed. G. Sato. New York: Plenum Press. p. 87.

BLUME, A., GILBERT, F., WILSON, S., FARBER, J., ROSENBERG, R., & NIRENBERG, M. (1970) Regulation of Acetylcholinesterase in Neuroblastoma Cells. *Proc. natn. Acad. Sci.*, **67**, 786.

BONDY, S. C., PRASAD, K. N. & PURDY, J. L. (1974) Neuroblastoma: Drug-induced Differentiation Increases Proportion of Cytoplasmic RNA that Contains Polyadenylic Acid. *Science N.Y.*, **186**, 259.

FURMANSKI, P., SILVERMAN, D. J. & LUBIN, M. (1971) Expression of Differentiated Functions in Mouse Neuroblastoma Mediated by Dibutyryl Cyclic Monophosphate. *Nature, Lond.*, **233**, 413.

GILMAN, A. G. & NIRENBERG, M. (1971) Regulation of Adenosine 3′5′-Cyclic Monophosphate Metabolism in Cultured Neuroblastoma Cells. *Nature, Lond.*, **234**, 356.

PRASAD, K. N. (1972) Cyclic AMP-Induced Differentiation Mouse Neuroblastoma Cells Lose Tumorgenic Characteristics. *Cytobios*, **6**, 163.

PRASAD, K. N. & HSIE, A. W. (1971) Morphological Differentiation of Mouse Neuroblastoma Cells Induced *in vitro* by Dibutyryl Adenosine, 3′:5′-cyclic Monophosphate. *Nature, New Biol.*, **233**, 141.

Prasad, K. N. & Kumar, S. K. (1974) Cyclic AMP and the Differentiation of Neuroblastoma Cells. In *Control of Proliferation in Animal Cells.* Eds. B. Clarkson and R. Baserga, Cold Spring Harbor: Cold Spring Harbor Laboratory, 581.

Prasad, K. N., Kumar, S., Gilmer, D. & Vernadakis, A. (1973) Cyclic AMP-Induced Differentiated Neuroblastoma Cells: Changes in Total Nucleic Acid and Protein Contents. *Biochem. biophys. Res. Commun.*, **50**, 973.

Prasad, K. N. & Mandal, B. (1973) Choline Acetyltransferase Level in Cyclic AMP and X-ray Induced Morphologically Differentiated Neuroblastoma Cells in Culture. *Cytobios*, **8**, 75.

Prasad, K. N. & Sheppard, J. R. (1972) Neuroblastoma Cell Cultures: Membrane Changes During Cyclic AMP-Induced Morphological Differentiation. *Proc. Soc. exp. Biol. Med.*, **141**, 240.

Prasad, R., Prasad, N. & Prasad, K. N. (1973) Esterase, Malate and Lactate Dehydrogenase Activity in Murine Neuroblastoma. *Science, N.Y.*, **181**, 450.

Prasad, R., Prasad, N. & Tevethia, S. S. (1972) Expression of Lactate and Malate Dehydrogenase in Tumors Induced by SV-40 and 7, 12-Dimethylbenz(a)-anthracene. *Science, N.Y.*, **178**, 70.

Prasad, R., Romsdahl, M. M., Shaw, C., R. Mumford, D. M. & Smith, J. L. (1974) Isozyme Variation in Human Malignant Melanoma. *Cancer Res.*, **34**, 1435.

Richelson, E. (1973) Stimulation of Tyrosine Hydroxylase Activity in an Adrenergic Clone of Mouse Neuroblastoma by Dibutyryl Cyclic AMP. *Nature, New Biol.*, **242**, 175.

Shaw, C. R. & Prasad, R. (1970) Starch Gel Electrophoresis of Enzymes. A Compilation of Recipes. *Biochem. Genet.*, **4**, 297.

Sheppard, H., Wiggan, G. & Tsien, W. H. (1972) Structure–Activity Relationships for Inhibitors of Phosphodiesterase from Erythrocytes and other Tissue. In *Advances in Cyclic Nucleotide Research.* Eds. P. Greengard, G. A. Robison & R. Paolette. New York: Raven Press. p. 102.

Waymire, J. C., Weiner, N. & Prasad, K. N. (1972) Regulation of Tyrosine Hydroxylase Activity in Cultured Mouse Neuroblastoma Cells: Elevation Induced by Analogs of Adenosine 3′ : 5′-Cyclic Monophosphate. *Proc. natn. Acad. Sci., USA*, **69**, 2241.

Wolf, U. & Engel, W. (1972) Gene Activation during Early Development of Mammals. *Humangenetik*, **15**, 99.


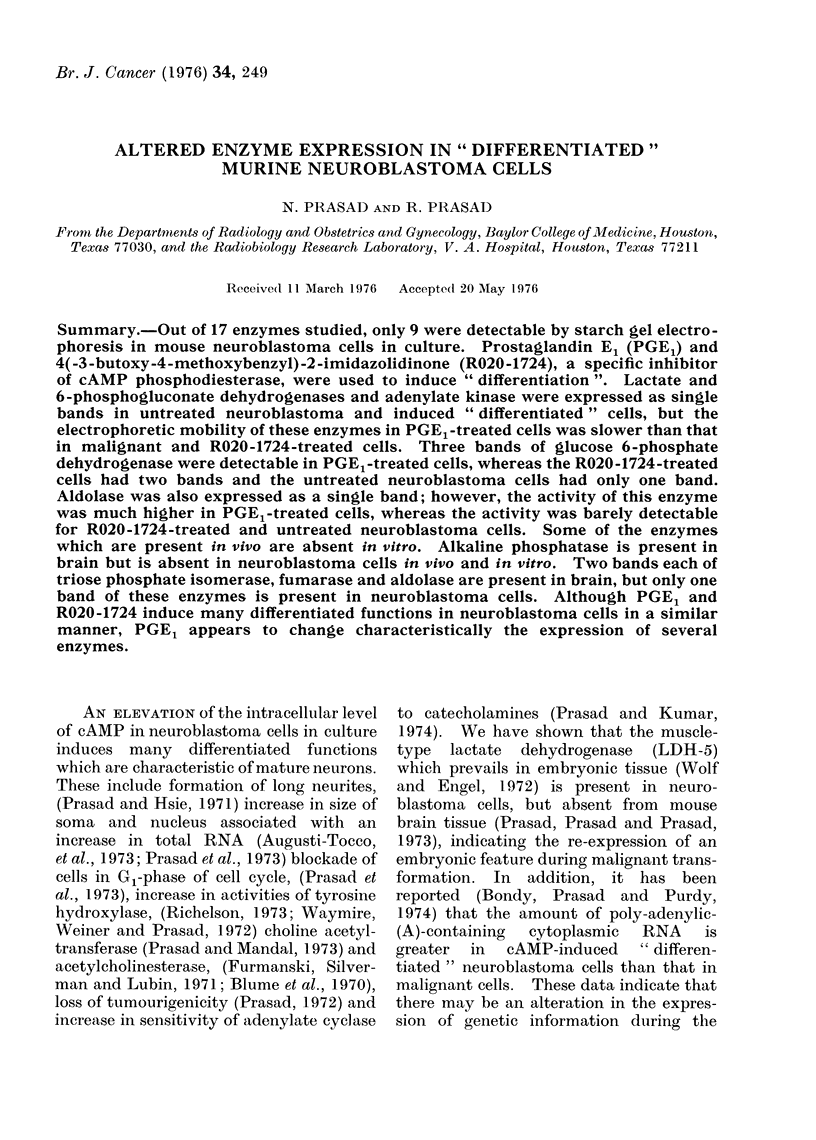

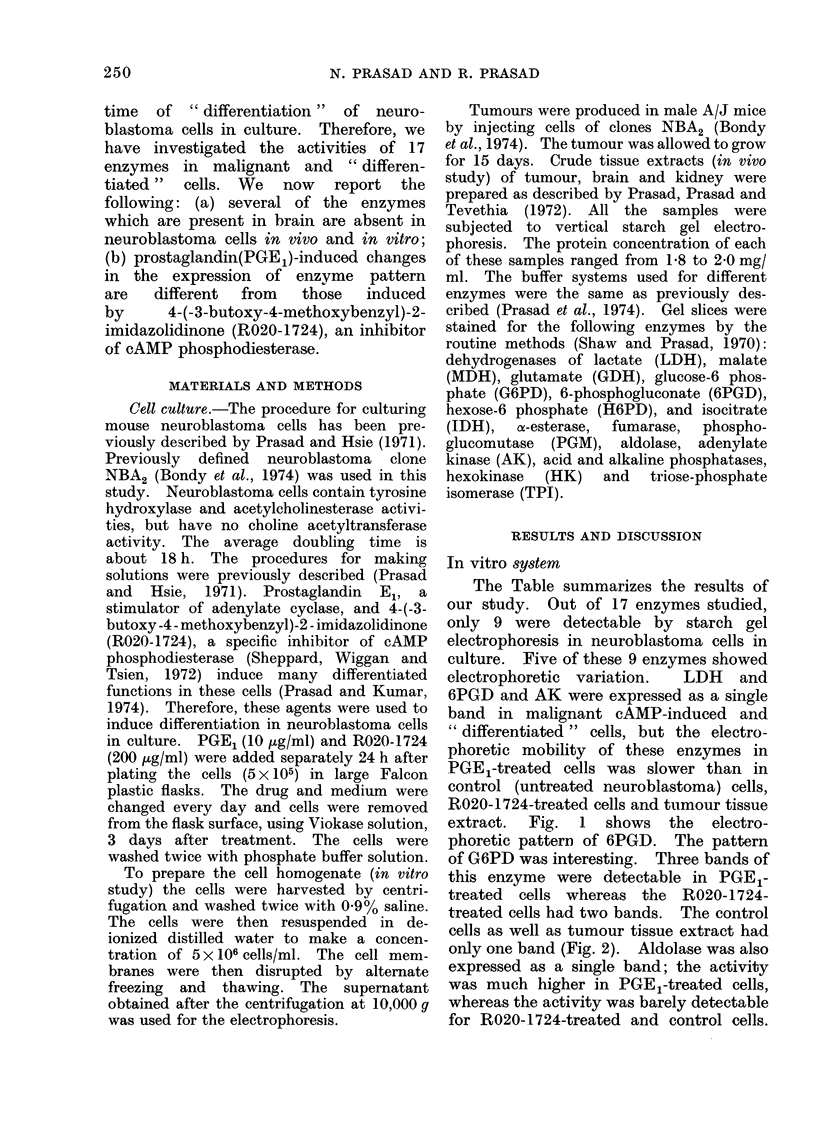

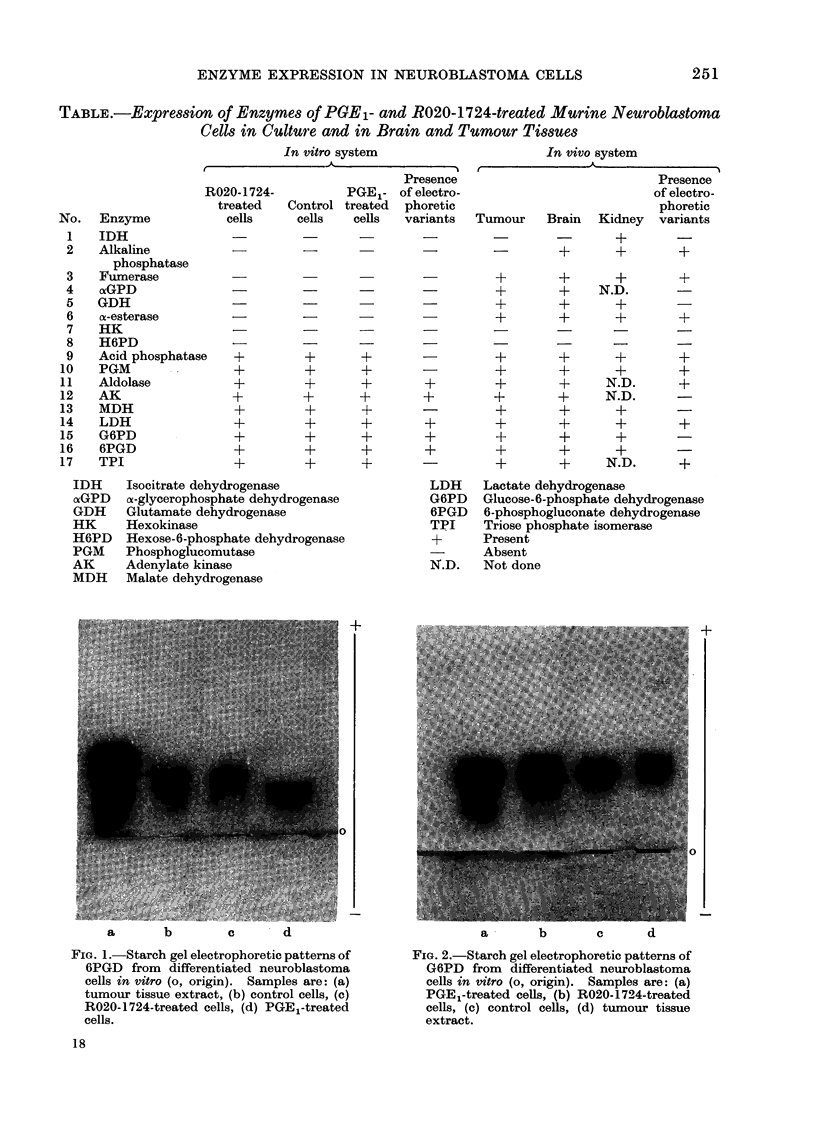

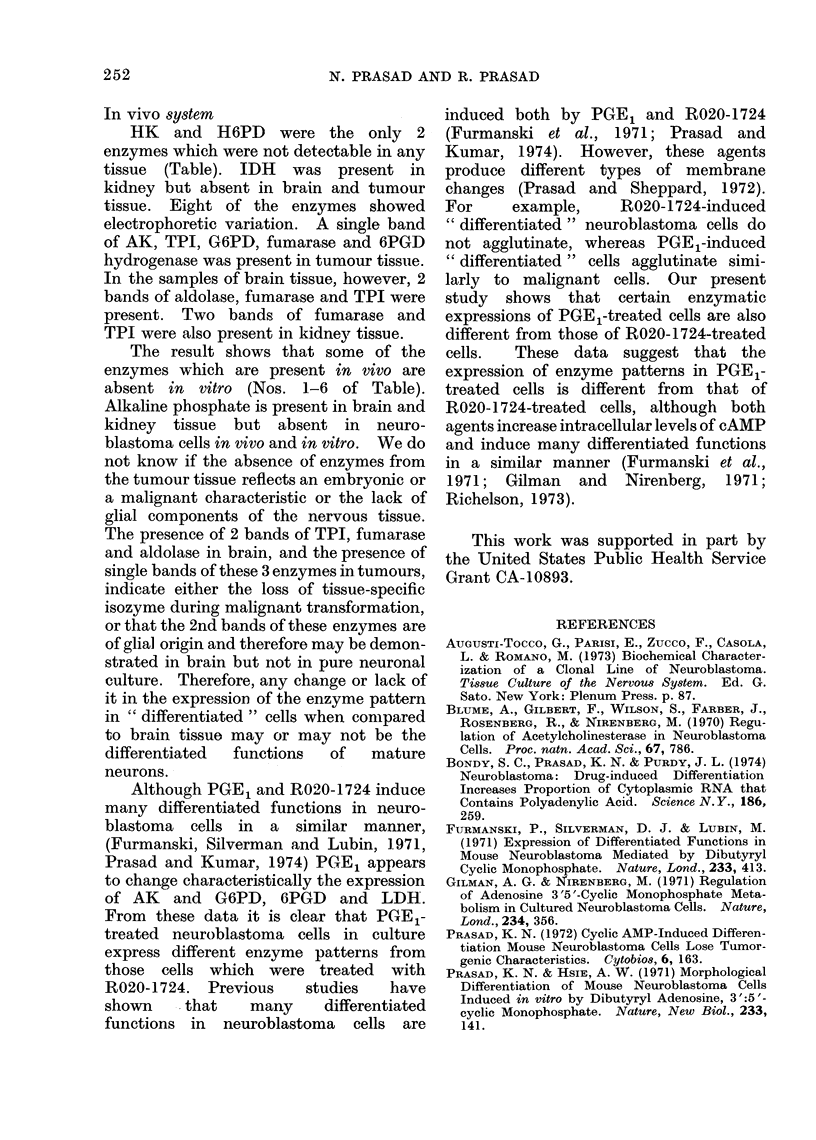

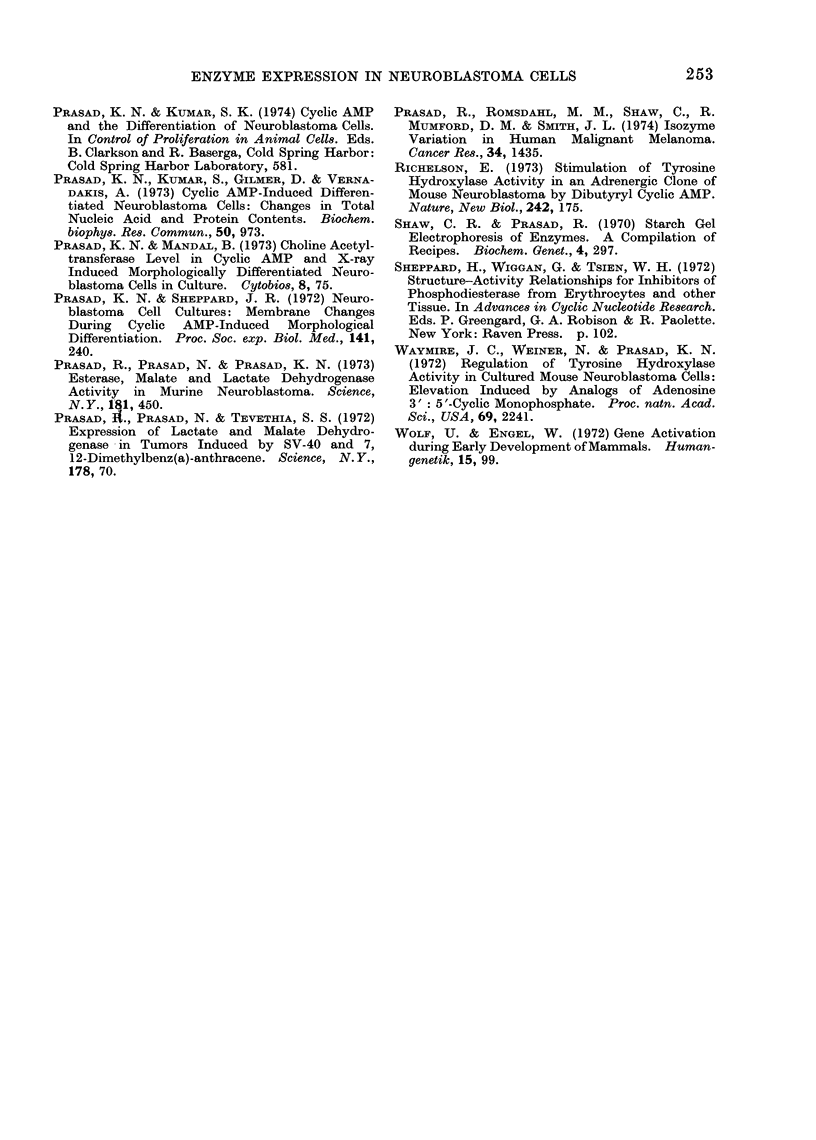


## References

[OCR_00495] Blume A., Gilbert F., Wilson S., Farber J., Rosenberg R., Nirenberg M. (1970). Regulation of acetylcholinesterase in neuroblastoma cells.. Proc Natl Acad Sci U S A.

[OCR_00508] Furmanski P., Silverman D. J., Lubin M. (1971). Expression of differentiated functions in mouse neuroblastoma mediated by dibutyryl-cyclic adenosine monophosphate.. Nature.

[OCR_00513] Gilman A. G., Nirenberg M. (1971). Regulation of adenosine 3',5'-cyclic monophosphate metabolism in cultured neuroblastoma cells.. Nature.

[OCR_00519] Prasad K. N. (1972). Cyclic AMP-induced differentiated mouse neuroblastoma cells lose tumourgenic characteristics.. Cytobios.

[OCR_00524] Prasad K. N., Hsie A. W. (1971). Morphologic differentiation of mouse neuroblastoma cells induced in vitro by dibutyryl adenosine 3':5'-cyclic monophosphate.. Nat New Biol.

[OCR_00547] Prasad K. N., Mandal B. (1973). Choline acetyltransferase level in cyclic AMP and x-ray induced morphologically differentiated neuroblastoma cells in culture.. Cytobios.

[OCR_00553] Prasad K. N., Sheppard J. R. (1972). Neuroblastoma cell culture: membrane changes during cyclic AMP-induced morphological differentiation.. Proc Soc Exp Biol Med.

[OCR_00560] Prasad R., Prasad N., Prasad K. N. (1973). Esterase, malate, and lactate dehydrogenases activity in murine neuroblastoma.. Science.

[OCR_00575] Prasad R., Romsdahl M. M., Shaw C. R., Mumford D. M., Smith J. L. (1974). Isozyme variations in human malignant melanoma.. Cancer Res.

[OCR_00579] Richelson E. (1973). Stimulation of tyrosine hydroxylase activity in an adrenergic clone of mouse neuroblastoma by dibutyryl cyclic AMP.. Nat New Biol.

[OCR_00585] Shaw C. R., Prasad R. (1970). Starch gel electrophoresis of enzymes--a compilation of recipes.. Biochem Genet.

[OCR_00598] Waymire J. C., Weiner N., Prasad K. N. (1972). Regulation of tyrosine hydroxylase activity in cultured mouse neuroblastoma cells: elevation induced by analogs of adenosine 3':5'-cyclic monophosphate.. Proc Natl Acad Sci U S A.

[OCR_00606] Wolf U., Engel W. (1972). Gene activation during early development of mammals.. Humangenetik.

